# P-2017. A Clinical Management Algorithm Using Society for Vascular Surgery Wound, Ischemia, and Foot Infection (WIfI) Score in Emergency Departments (ED) for Lower Extremity Wounds (LEW) Improves Diagnostic Efficiency and Resource Allocation, Implications for Quality Improvement (QI)

**DOI:** 10.1093/ofid/ofaf695.2181

**Published:** 2026-01-11

**Authors:** Colin Samoriski, Christine Kim, James D Brodell, Joseph A O’Loughlin, Justin Hopkin, Ted Louie, Alexandra Yamshchikov

**Affiliations:** University of Rochester, Rochester, NY; University of Rochester Medical Center, Rochester, New York; University of Rochester Medical Center, Rochester, New York; University of Rochester Medical Center, Rochester, New York; University of Rochester, Rochester, NY; University of Rochester Medical Center, Rochester, New York; University of Rochester School of Medicine and Dentistry, Rochester, NY

## Abstract

**Background:**

Management of diabetic and nondiabetic LEW is often associated with diagnostic uncertainty and inconsistent resource utilization. A comprehensive clinical management algorithm is challenging to develop and implement.

**Figure 1.**
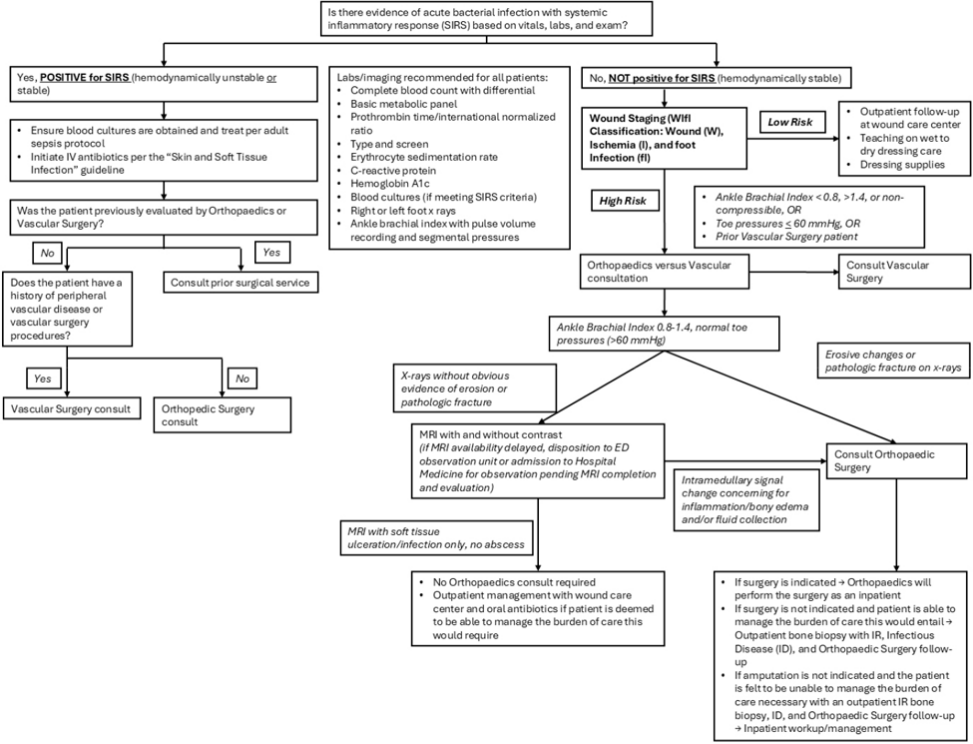
Guideline for Triage of Adults with Lower Extremity Wound (LEW) Institutional clinical practice guideline developed for the evaluation and management of patients presenting to the ED with lower extremity wound implemented at the intervention hospital.

**Table 1.**
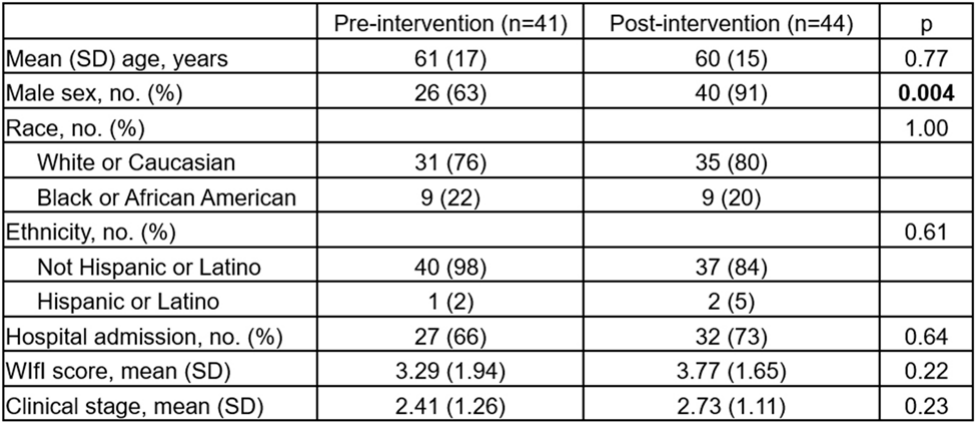
Demographic and Baseline Characteristics

**Methods:**

A multidisciplinary QI group implemented institutional clinical practice guidelines for management of LEW inclusive of WIfI score (Fig. 1) to facilitate workup and determine admission status at 1 of 7 network hospitals in a large academic healthcare system. An EHR SQL report using custom SNOMED CT concept hierarchy grouper of 1217 ICD-10 codes identified triage and process metrics for 85 patients presenting to network hospital EDs for evaluation of LEW within 3 months pre- (n=41; 12 intervention, 29 control patients) and post-implementation (n=44; 18 intervention, 26 control patients), separated by 1 month washout. WIfI scores and clinical stage (1=very low risk to 4=high risk) were assigned according to published methods, based on clinical information at presentation. Process outcomes at the intervention hospital were compared to non-intervention hospitals using t-test or Fisher’s exact test.

**Table 2.**
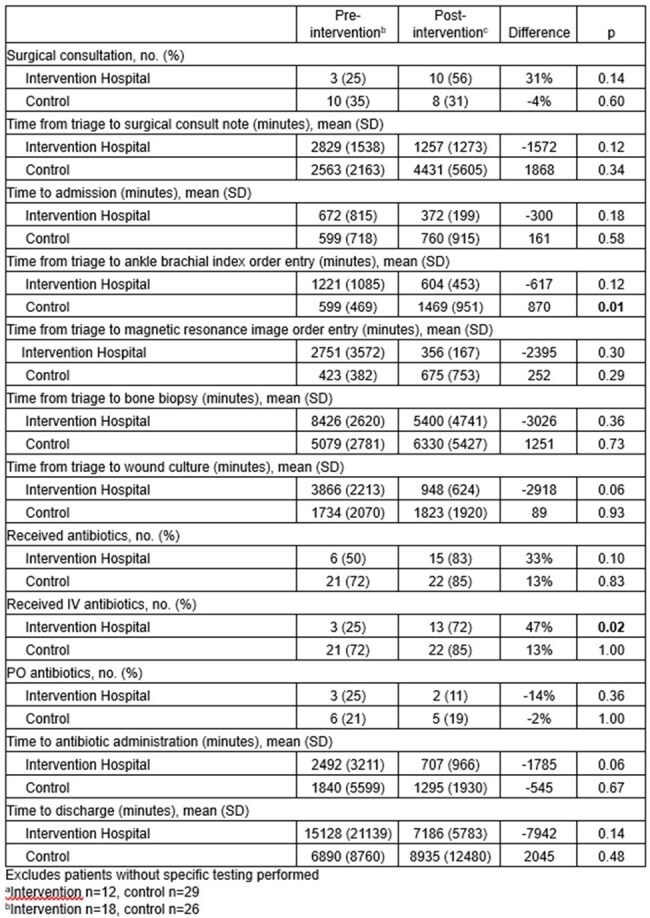
Outcomes and Process Measures

**Figure 2.**
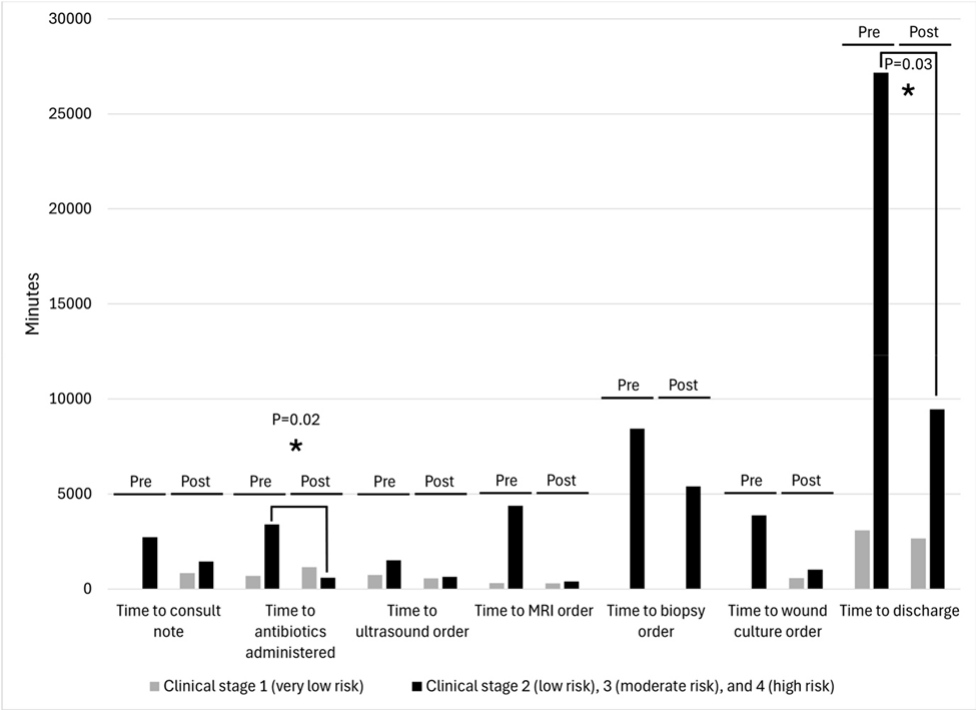
Mean Time to Outcome Measures Pre- and Post-Intervention by Clinical Stage Based on WIfI score

**Results:**

Baseline characteristics between pre- and post-implementation cohorts were similar except gender post-intervention (Tab. 1). Frequency of surgical consultation increased and time to consult completion decreased (Tab. 2), as did time to inpatient admission, ankle brachial index and MRI orders, and time to wound culture and bone biopsy, though differences were not statistically significant. Overall and intravenous antibiotic use in the intervention hospital increased by 33% (P=0.10) and 47% (P=0.02), respectively. Time to discharge fell by 132 hours (P=0.14) in the intervention hospital and increased by 34 hours at control hospitals (P=0.48). Improvement was most pronounced in patients with higher clinical stage (Fig. 2).

**Conclusion:**

A novel clinical management algorithm using a WIfI score for triage and workup decisions for LEW was successfully implemented at a large academic hospital, with trends toward improvement in clinical process metrics. Although comparison with non-intervention hospitals suggests meaningful improvement in efficiency and resource allocation, larger studies are needed.

**Disclosures:**

Justin Hopkin, MD, Sanofi: I'm an emeritus board member for a national patient foundation. I receive travel/lodging to speak at 2 patient advocacy meetings.

